# Long-Term Effects from Bacterial Meningitis in Childhood and Adolescence on Postural Control

**DOI:** 10.1371/journal.pone.0112016

**Published:** 2014-11-18

**Authors:** Hannes Petersen, Mitesh Patel, Einar F. Ingason, Einar J. Einarsson, Ásgeir Haraldsson, Per-Anders Fransson

**Affiliations:** 1 Department of Otorhinolaryngology, Landspitali University Hospital, Reykjavik, Iceland; 2 Faculty of Medicine, University of Iceland, Reykjavík, Iceland; 3 Children’s Hospital Iceland, Landspitali University Hospital, Reykjavik, Iceland; 4 Department of Clinical Sciences, Lund University, Lund, Sweden; 5 Department of Clinical Neuroscience, Neuro-Otology Department, Imperial College London, London, United Kingdom; University Children’s Hospital Tuebingen, Germany

## Abstract

Bacterial meningitis in childhood is associated with cognitive deficiencies, sensorimotor impairments and motor dysfunction later in life. However, the long-term effects on postural control is largely unknown, e.g., whether meningitis subjects as adults fully can utilize visual information and adaptation to enhance stability. Thirty-six subjects (20 women, mean age 19.3 years) treated in childhood or adolescence for bacterial meningitis, and 25 controls (13 women, mean age 25.1 years) performed posturography with eyes open and closed under unperturbed and perturbed standing. The meningitis subjects were screened for subjective vertigo symptoms using a questionnaire, clinically tested with headshake and head thrust test, as well as their hearing was evaluated. Meningitis subjects were significantly more unstable than controls during unperturbed (p≤0.014) and perturbed standing, though while perturbed only with eyes open in anteroposterior direction (p = 0.034) whereas in lateral direction both with eyes open and closed (p<0.001). Meningitis subjects had poorer adaption ability to balance perturbations especially with eyes open, and they frequently reported symptoms of unsteadiness (88% of the subjects) and dizziness (81%), which was found significantly correlated to objectively decreased stability. Out of the 36 subjects only 3 had unilateral hearing impairment. Hence, survivors of childhood bacterial meningitis may suffer long-term disorders affecting postural control, and would greatly benefit if these common late effects became generally known so treatments can be developed and applied.

## Introduction

Bacterial meningitis is associated with a significant mortality rate, that range from 4% in infants and children [1] to 20–30% in neonates and adults [2]. Preventive measures including immunization during the last decades have decreased the number of childhood meningitis [3], [4], however, follow-up of survivors is important. Symptoms commonly associated with bacterial meningitis include acute onset of high fever, headache, neck stiffness, photophobia and confusion. The survivors are at high risk to develop long-term sequelae despite appropriate antibiotic therapy and availability of vaccines [5]. The pathogenic reaction from meningitis can affect the inner ear [6] or lead to neuronal injury that includes cortical necrosis and hippocampal apoptosis [7]. Even after initial antibiotic therapy, secondary brain injury continues for up to four days due to inflammatory responses and a strong immune reaction [8]. Hence, bacterial meningitis is associated with learning and memory deficits, cognitive deficiencies and sensorimotor impairments including hearing and visual loss, and motor dysfunction [9]–[11]. Up to a third of all survivors suffer transient or permanent deafness or other neurological sequelae [12]. Some twenty percent of all childhood survivors experience neurological deficits at least five years after the initial diagnosis [9]. In addition to the neurological damage, ossification of the inner ear has been described, contributing to the long-term effects of childhood meningitis [11].

Postural control in humans requires continuous integration of sensory inputs from visual, vestibular and somatosensory receptors (proprioceptors and mechanoreceptors) to assess position and motion of the body [13]. Impairments of postural control as well as impairments of the sensory and motor systems in the central nervous system (CNS) may be expressed as decreased stability or disturbed movement control. However, even individuals with balance deficits are often able to handle less postural demanding conditions like quiet stance reasonably well. This limits the chances that assessments of quiet stance stability using posturography can discern and classify balance impairments [14]. To increase the sensitivity of posturography to reveal balance deficits one therefore commonly uses balance perturbations to strain the stability control, e.g. by disrupting the somatosensory information by applying vibration against muscles or tendons important for standing [15]. Such vibration simultaneously increases the afferent signals from the muscle spindles and creates a proprioceptive illusion that the vibrated muscle is being stretched. The responses subsequently induced are intended to return the vibrated muscle to its perceived original length [16], for a detailed graphical illustration of the typical stimulus-response pattern, see figure 2 in [17]. Calf muscle vibration typically increases movements bi-directionally, though generally more in anteroposterior direction than in lateral direction [18].

When balance perturbations are repeated, a healthy individual is quickly able to learn how to adjust to the new demands and handle the task better, a process termed adaptation. Human postural control adaptation involves recalibration of motor programs, sensorimotor pathways and strategies, such as changes of the body movement pattern [19], [20]. Adaptation in standing minimizes costs including energy demands, forces, fatigue, inaccuracy, jerkiness etc. Thus, assessment of adaptation capacity can help to identify subtle impairments of the CNS and sensorimotor systems [20]–[23], an option often overlooked in clinical assessments. CNS disorders such as damage to the cerebellum often impairs adaptation [23]. How the adaptation processes are affected by meningitis infection in childhood is to date unknown.

To date, little is known about the long-term effects of bacterial meningitis in childhood and adolescence on human postural control and about the CNS remaining ability to utilize visual information and adaptation to enhance stability. Verified long-term impairment of postural control would add to growing evidence that bacterial meningitis at early age may cause a multitude of long-lasting CNS deficits. The study objective was to evaluate postural control and adaptation capacity during sustained sensorimotor challenges in survivors of childhood meningitis. In addition, the meningitis subjects were screened using a questionnaire to assess subjective symptoms related to decreased stability such as feeling dizziness, visual impairments and vertigo symptoms.

## Materials and Methods

### Ethics Statement

The experiments were performed in accordance with the Helsinki declaration and approved by the Scientific Ethical Committee number VSNb2010110017/03.7 and The Data Protection Authority number 2010111027AT in Iceland. All participants or their guardians, provided written informed consent before the testing commenced.

### Subjects

All children and adolescents between 0.5–18 years of age diagnosed with bacterial meningitis during the years 1990–2010 at Landspitali University Hospital, Iceland and with treatment completed at least 1 year before this study, were included. The diagnosis of bacterial meningitis was confirmed with cerebral spinal fluid (CSF) analysis, bacterial culture, PCR antigen detection of CSF or positive blood cultures. The subjects were screened using a questionnaire addressing any medical reasons that might exclude them such as serious injuries involving their lower extremities, major CNS-trauma or neurological deficits that could cause vertigo or balance problems but were of other origin than that of a meningitis infection. Furthermore, all subjects were clinically evaluated with headshake and head thrust tests.

The study group consisted of 36 subjects, 20 women and 16 men of mean age 19.3 years (SD 4.2 years, range 14–28 years); mean height 1.73 meters (SD 0.11 m); mean weight 78.9 kilograms (SD 19.0 kg). Both height and weight were within validated limits of the balance platform used. The mean age at diagnosis was 6.8 years (SD 6.1 years, range 0.6–17.0 years) and only one child was younger than 12 months when diagnosed. The mean time since the end of meningitis treatment was 12.5 years (SD 5.0 years, range 1.0–20.3 years) and aminoglycosides were not included in the treatment protocols. The healthy, age-matched, control group consisted of 25 participants, 13 women and 12 men of mean age 25.1 years (SD 4.6 years, range 19–41 years); mean height 1.75 meters (SD 0.09 m); mean mass 68.8 kilograms (SD 13.3 kg).

### Posturography assessment

The posturography was performed using a validated test paradigm [15], [16], [25], where human postural control was evaluated by recording body sway during quiet stance and proprioceptive balance disturbances. In comparison to the Equitest MSOT test paradigm [26] the longer test durations of 230 seconds allows assessments of regulating processes associated with low frequency stability and the efficiency of the stability enhancing adaptive processes, which in healthy individuals is manifested as an over-time augmented capacity to handle new demands and task [19], [20]. Additionally, the analysis procedures of the posturography test paradigm ensures that all calculated values are properly normalized for the subjects anthropometrical variations both in height and mass.

A custom built force platform recorded torques and sheer forces with six degrees of freedom using force transducers with an accuracy of 0.5 N. A customized computer program controlled the vibratory stimulation and sampled the force platform data at 50 Hz. The vibrators had vibration amplitude of 1.0 mm and frequency of 85 Hz, were 6 cm long and 1 cm in diameter and strapped over the calf muscles of both legs.

Each participant stood barefoot on the force platform in a relaxed posture with arms folded across the chest, heels 3 cm apart and feet positioned at an angle of 30° along guidelines on the platform. Participants were instructed to focus on a target 1.5 m in front of them at eye level or keep their eyes closed depending on the test condition. The participants listened to music through headphones to reduce possible movement references from external noise sources and to avoid extraneous sound distractions. To ensure no prediction of the balance perturbations, all participants were naive to the stimulus and were not informed about the effect calf vibration would have on their balance. To protect the subjects from falling, an individual was assigned to observe the subjects throughout the test procedure.

Two tests were performed in a randomized order, using a Latin Square design, by all subjects: 1) Vibration of the calf muscles with eyes closed (EC) and 2) Vibration of the calf muscles with eyes open (EO).

Before vibration commenced, a 30 second control period of quiet stance was recorded. The test paradigm vibratory stimulation disturb only somatosensory information originating from a controlled limited local area [15]. The stimulus sequence is implemented to evaluate within a broad spectral range the robustness of the subject’s postural control while maintaining the same amplitude properties of the somatosensory distractions [14]. The vibratory stimulations were applied according to a pseudorandom binary sequence (PRBS) schedule during a period of 200 seconds making each trial 230 seconds long. The PRBS schedule defined the periodicity of stimulation pulses, where each pulse and each interval between pulses had random time duration from 0.8 seconds up to 6.4 seconds, which yielded an FFT-validated effective bandwidth of the test stimulus in the region of 0.1–2.5 Hz. The PRBS sequence was selected because this randomized stimulation sequence is difficult to predict and therefore lessens the likelihood of pre-emptive responses. An identical PRBS sequence was applied to all subjects during all test and the stimuli was simultaneously applied to the calf muscles of both legs. A five minute rest period was given between eyes open EO and EC tests.

### Hearing assessment

Pure-tone hearing thresholds were assessed for eight frequencies: 0.25, 0.5, 1, 2, 3, 4, 6, and 8 kHz, according to the Hughson–Westlake method. Pure-tone average (PTA) of thresholds at 0.5, 1, 2, 4 kHz (PTA_5124_) ≤20 dB hearing level (HL), was considered within an age-independent normal limit. Madsen-Aurical audiometer and TDH39P (Telephonics) audiometric headset were used.

Tympanometry was performed to test the middle ear pressure and mobility of the tympanic membrane and the middle ear ossicles. Middle ear pressure between −100 and +50 daPa and compliance between 0.3 and 1.6 ml were defined as normal and scored according to Jerger’s classification. Madsen-OTOflex 100 was used.

Distortion Products Otoacoustic Emissions (DPOAE) was used to measure outer hair cell function of the cochlea, using Madsen-Capella cochlear emissions analyzer. DPOAE was obtained by using two simultaneous pure-tone signals (primaries) of two different frequencies: f1 and f2, of different amplitudes with fixed frequency ratio f2/f1 at 1.22. The amplitude of the response frequency component at 2f1–f2, resulting from cubic distortion product was recorded. Levels of the primaries were set to 65 dB SPL (peak), for primary 1 (f1) and 55 dB SPL (peak), for primary 2 (f2). The criterion for acceptable DPOAE response was set to a signal to noise ratio (S/N) of ≥3 dB for each measurement (i.e., the response was double the noise amplitude).

### VSS questionnaire

Data regarding the level of experienced handicap were gathered by means of The Vertigo Symptom Scale (VSS) in an Icelandic translation. This questionnaire has been found to discriminate well between patients complaining of dizziness and healthy controls and in tests of reliability and validity [27], [28]. The questionnaire is customized to target problems with dizziness, imbalance and vision. The form consists of 22 questions and the participants had to answer all of them. All subjective symptoms were rated by how often the subject had felt these symptoms during the last year: 0) never, 1) 1–3 times a year, 2) 4–12 times a year, 3) more than once a month and 4) more than once a week. Three questions (20, 21 and 22), categorize the type of dizziness felt as: things are spinning or moving around; feeling of being light-headed and feeling unsteady. For these three questions, the symptoms were described in detail by asking for the duration of experiencing these symptoms and by grouping the answers into four subcategories: a) less than 2 min, b) 2–60 min, c) more than 1 hour and d) the whole day. Each of these four subgroups were also rated by how often the subject had felt these symptoms during the last year giving scores of 0–4 points. The frequency of experiencing symptoms was considered in the correlation analyses between questionnaire answers and recorded stability, described below, by using the above frequency scores of 0–4 points for each person and question.

### Analysis

Postural stability was measured by a force platform as the variance of anteroposterior and lateral torque values used towards the support surface. Recorded torque contain the same information about movement fluctuations as the traditional method of calculating CoP. However, the information is presented in the form of energy used towards the support surface to maintain stability [21], [29], [30], which in turn corresponds to the efficiency of standing [31]. Moreover, the values are always normalized before statistical analysis for anthropometrical variations in height and mass. The force platform recordings were spectrally divided into total torque variance, torque below 0.1 Hz (<0.1 Hz; low frequency); and torque above 0.1 Hz (>0.1 Hz; high frequency) using a fifth-order digital Finite duration Impulse Response (FIR) filter, with filter components selected to avoid aliasing. These separations were used to distinguish better between smooth corrective changes of posture (i.e. <0.1 Hz) and fast corrective largely reflexive movements made to maintain balance (i.e. >0.1 Hz) [32]. Torque variance values were normalized to account for anthropometric differences between the subjects, using the subject’s squared height and squared mass, as height and mass are key factors influencing the body sway recorded by a force platform [21], [29]. The squared nature of the variance algorithm made it necessary to use normalization with squared parameters to achieve unit agreement.

Mean values for all parameters were obtained for five periods for each trial condition: the quiet stance period (0–30 s), and from four 50 s periods (period 1: 30–80 s; period 2: 80–130 s; period 3: 130–180 s; period 4: 180–230 s) during the vibration. Each 50 s period contains a similar amount of long and short vibration pulses validated by Fast-Fourier Transform (FFT)-analysis of spectral contents in the stimulation. Hence, the selected periods and perturbation sequence allowed analysis of whether the stability changed over time and possibly caused an adaptation to the unpredictable balance perturbations.

### Statistical analysis

The torque variance values during quiet (unperturbed) stance and during balance perturbations were analyzed using repeated measures GLM ANOVA on log-transformed values. The log-transformation made prior to the statistical analysis was done to reduce the non-normal distribution of the data sets, produced by that the variance algorithm used in the data analysis include calculating the sum of squared values. The main factors and factor interactions analyzed were: The effects of having had meningitis in childhood (‘Meningitis’: Yes or No; (1 degree of freedom (d.f.)); availability of visual information (‘Vision’: eyes closed or eyes open; d.f. 1), and when applicable the period of vibration (‘Period’: periods 1–4; d.f. 3).

The Mann-Whitney test was used for post hoc comparison between meningitis treated subjects and the healthy controls. Wilcoxon matched-pairs signed-rank test (Exact sig. 2-tailed) was used for analysis of adaptive changes over time for both groups between period 1 and period 4 [21], [30].

Correlations evaluating relationships between: a) age at time of meningitis treatment; b) time elapsed between end of treatment and this study assessment; c) answers in the questionnaire and d) recorded total torque variance values, was made using the Spearman’s Rank correlation analysis.

In all analyses, p-values<0.05 were considered statistically significant. In the pair-wise comparisons, no data sets were included in a data analysis more than once, meaning no Bonferroni correction was necessary. Non-parametric statistical tests were used in all statistical evaluations since the Shapiro-Wilk test revealed that some of the obtained data sets were not normally distributed.

## Results

### Quiet stance stability analyzed by GLM ANOVA

The quiet stance stability in anteroposterior direction was poorer in meningitis subjects compared with controls, as reflected by the significantly increased total (p = 0.014, +58%) and low frequency (p = 0.010, +75%) torque variance, found related to the “Meningitis” factor in the analysis (Table 1). Visual information, denoted as factor “Vision”, markedly improved the stability in both subject groups, i.e. reduced significantly total (p<0.001, −28%) and high frequency (p<0.001, −38%) torque variance. Furthermore, the significant interaction between the factors “Meningitis” and “Vision” showed that the stability with eyes open was poorer in meningitis subjects compared with controls, as reflected by the increased total (p = 0.028; +87% larger than controls with eyes open vs. +29% larger with eyes closed) and low frequency torque variance (p = 0.013; +131% with eyes open vs. +19% with eyes closed) (Table 1).

**Table 1:** Statistical evaluation of anthropometrical height and mass normalized torque variance values during quiet stance, comparing subjects treated in childhood for meningitis with healthy controls.

Normalized Torque variance		p-values*		
Quiet stance		Meningitis	Vision	Meningitis×Vision
Anteroposterior	Total	**0.014 [6.4]**	**<0.001 [12.3]**	**0.028 [5.1]**
	<0.1 Hz	**0.010 [7.1]**	0.378 [0.8]	**0.013 [6.6]**
	>0.1 Hz	0.179 [1.9]	**<0.001 [42.6]**	0.792 [0.1]
Lateral	Total	**<0.001 [40.9]**	0.253 [1.3]	0.544 [0.4]
	<0.1 Hz	**<0.001 [20.4]**	0.585 [0.3]	0.676 [0.2]
	>0.1 Hz	**<0.001 [43.4]**	***0.054 [3.9]***	0.531 [0.4]

*The notation “<0.001” means that the p-value is smaller than 0.001. Values in bold show p-values<0.05 and values in bold italic show p-values<0.1. F-values are presented within the squared parenthesis.

The quiet stance stability in lateral direction was poorer in meningitis subjects compared with controls, reflected by the significantly increased torque variance in all spectral categories found related to the “Meningitis” factor in the analysis (p<0.001; total +164%, low +151%, high +178%) (Table 1). The factor “Vision” indicated that visual information had no significant influence on the stability in lateral direction. Moreover, no significant interaction was found between the factors “Meningitis” and “Vision”.

**Figure 1:**
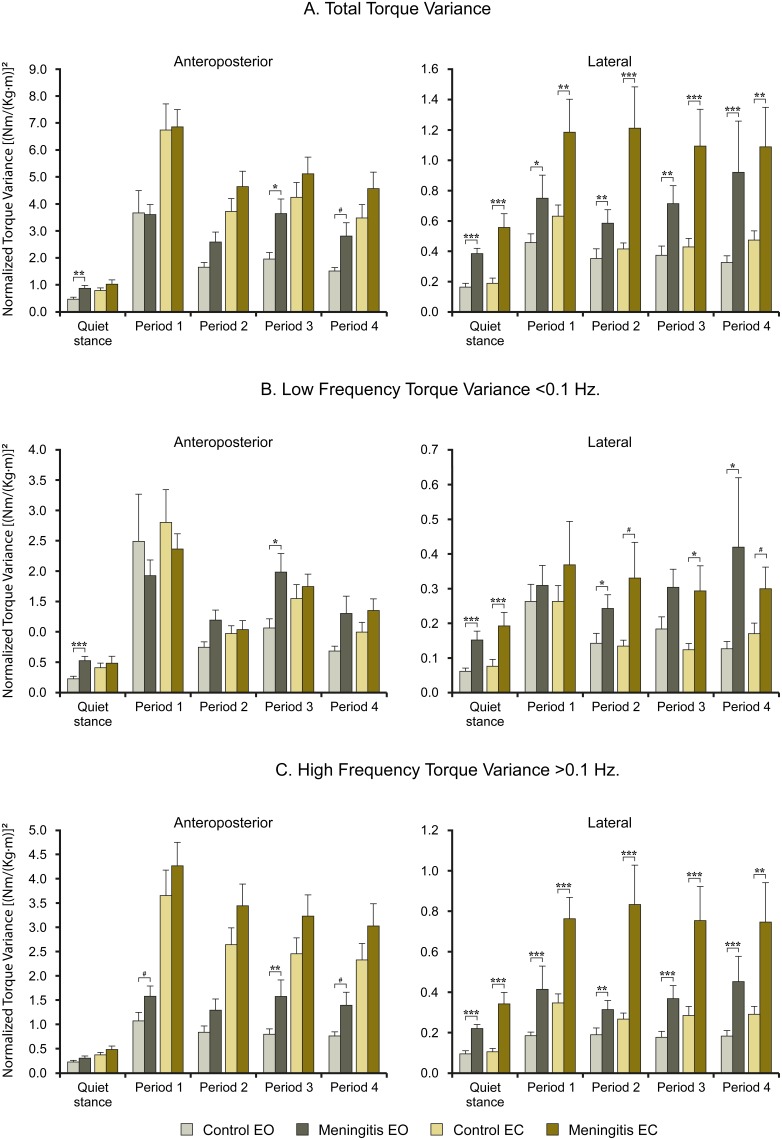
Anthropometrical height and mass normalized values for (A) total, (B) low frequency and (C) high frequency torque variance with Eyes Closed and Eyes Open (mean and SEM) for healthy controls (n = 25) and for meningitis treated subjects (n = 36). The figures present the statistical findings made in the post hoc evaluation of the main factor meningitis. #denotes p<0.1 (trends), *denotes *p*<0.05, **denotes *p*<0.01 and ***denotes *p*<0.001.

Post-hoc data analysis of individual tests confirmed that the quiet stance stability were generally poorer in meningitis subjects compared with controls both in anteroposterior and lateral directions in all spectral categories, both with eyes closed and eyes open, see Figure 1.

### Perturbed stance stability analyzed by GLM ANOVA

There were no significant differences in anteroposterior stability during balance perturbations between meningitis subjects and controls as revealed by the “Meningitis” factor in the analysis (Table 2). The factor “Vision” indicated that visual information markedly improved stability in both subject groups, i.e. significantly reduced total (p<0.001, −46%) and high frequency torque variance (p<0.001, −63%). The “Period” factor, evaluating stability changes over time, indicated that the repeated balance perturbations disturbed the stability significantly less over time in both subject groups in all spectral categories (p<0.001; total −41%, low −53%, high −27%). The interaction between the factors “Vision” and “Period” showed that the high frequency torque variance had significantly larger decline over time, i.e., the stability improved more, with eyes closed (−33%) than with eyes open (p = 0.007, −21%). Moreover, the significant interaction between the factors “Meningitis”, “Vision” and “Period” showed that meningitis subjects had larger low frequency torque variance than controls, i.e., poorer stability, while standing with eyes open during the latter part of the tests (p = 0.040; +91% larger than controls in period 4 with eyes open vs. +36% larger with eyes closed).

**Table 2:** Statistical evaluation of normalized torque variance values during balance perturbations, comparing subjects treated in childhood for meningitis with healthy controls.

Normalized Torque variance		p-values*					
Balance perturbations		Meningitis	Vision	Period	M×P	V×P	M×V×P
Anteroposterior	Total	***0.079 [3.2]***	**<0.001 [75.1]**	**<0.001 [56.9]**	***0.056 [3.8]***	0.428 [0.6]	0.124 [2.4]
	<0.1 Hz	0.203 [1.7]	***0.099 [2.8]***	**<0.001 [38.6]**	***0.083 [3.1]***	0.193 [1.7]	**0.040 [4.4]**
	>0.1 Hz	***0.073 [3.3]***	**<0.001 [142.4]**	**<0.001 [42.8]**	0.479 [0.5]	**0.007 [7.9]**	0.789 [0.1]
Lateral	Total	**<0.001 [15.4]**	**<0.001 [47.1]**	**<0.001 [17.9]**	**0.028 [5.1]**	***0.085 [3.1]***	0.182 [1.8]
	<0.1 Hz	**0.008 [7.4]**	0.757 [0.1]	**0.002 [10.1]**	***0.083 [3.1]***	0.136 [2.3]	0.675 [0.2]
	>0.1 Hz	**<0.001 [17.0]**	**<0.001 [111.4]**	**0.001 [12.0]**	0.312 [1.0]	**0.015 [6.3]**	**0.041 [4.4]**

*The interaction combination not shown in the table included no values close to significance or trends. In the table, M denotes main factor Meningitis, P = Period and V = Vision.

A further analysis of the effects of meningitis on anteroposterior stability, for eyes closed or eyes open (Table 3), revealed that with eyes closed, the stability in all spectral categories was less disturbed over time by the balance perturbations, as shown by the significant “Period” factor (p<0.001; total −41%, low −54%, high −33%). However with eyes open, the meningitis subjects had poorer stability compared with healthy controls as reflected by the significantly higher total (p = 0.034, +57%) and high frequency torque variance (p = 0.032, +71%) found related to the “Meningitis” factor. The “Period” factor revealed that the balance perturbations with eyes open disturbed the stability significantly less over time in all spectral categories (p≤0.005; total −40%, low −53%, high −21%). Furthermore, the interaction between the factors “Meningitis” and “Period”, showed that the meningitis subjects had significantly less total torque variance reduction (p<0.028), i.e., less stability improvement (−28%) over time compared to healthy controls (−54%).

**Table 3:** Statistical evaluation of normalized torque variance values during balance perturbations, investigating the performance separately with eyes closed and eyes open.

Normalized Torque variance		p-values*					
		Eyes Closed			Eyes Open		
Balance perturbations		Meningitis	Period	M×P	Meningitis	Period	M×P
Anteroposterior	Total	0.260 [1.3]	**<0.001 [43.7]**	0.430 [0.6]	**0.034 [4.7]**	**<0.001 [28.6]**	**0.028 [5.1]**
	<0.1 Hz	0.453 [0.6]	**<0.001 [21.6]**	0.326 [1.0]	0.128 [2.4]	**<0.001 [25.7]**	***0.067 [3.5]***
	>0.1 Hz	0.265 [1.3]	**<0.001 [44.0]**	0.544 [0.4]	**0.032 [4.8]**	**0.005 [8.3]**	0.579 [0.3]
Lateral	Total	**<0.001 [15.4]**	**<0.001 [25.5]**	**0.029 [5.0]**	**<0.001 [12.4]**	**0.004 [8.7]**	**0.027 [5.1]**
	<0.1 Hz	**0.017 [6.1]**	**0.002 [10.4]**	0.169 [1.9]	**0.014 [6.4]**	**0.024 [5.4]**	0.128 [2.4]
	>0.1 Hz	**<0.001 [17.4]**	**<0.001 [15.2]**	***0.051 [4.0]***	**<0.001 [13.4]**	**0.008 [7.6]**	0.113 [2.6]

*In the table, M denotes the main factor Meningitis and P = Period.

The lateral stability was significantly poorer in meningitis subjects compared with healthy controls during balance perturbations, as reflected by the significantly increased torque variance in all spectral categories, i.e. total (p<0.001, +121%), low (p = 0.008, +98%) and high (p<0.001, +137%), found related to the “Meningitis” factor. (Table 2). The factor “Vision” showed that visual information improved the lateral stability in both subject groups, as reflected by significantly reduced total (p<0.001, −28%) and high frequency torque variance (p<0.001, −44%). The significant “Period” factor for total (p<0.001, −10%), low (p = 0.002, −18%) and high frequency torque variance (p = 0.001, −3%), showed that the repeated balance perturbations disrupted the stability significantly less over time in both subject groups.

The significant interaction between the factors “Meningitis” and “Period” for the total torque variance (p = 0.028) showed that the meningitis subjects had a poorer lateral stability improvement (+7%) over time compared to controls (−27%). The significant interaction between the factors “Vision” and “Period” revealed that the high frequency torque variance had a larger decline, i.e., better stability improvement over time with eyes closed (−9%) than with eyes open (+4%) (p = 0.015). Moreover, the significant interaction between the factors “Meningitis”, “Vision” and “Period” showed that the meningitis subjects initially had much larger high frequency torque variance, i.e., poorer stability, than controls while standing with eyes closed than during the latter part of the tests (p = 0.041; +213% larger than controls with eyes closed vs. +65% larger with eyes open in period 2 that changed to +158% with eyes closed vs. +148% with eyes open in period 4).

A further analysis of the effects of meningitis on lateral stability, for eyes closed or eyes open, revealed that meningitis had similar effects on the lateral stability during balance perturbations, regardless of eyes open or closed (Table 3). Both with eyes closed (p≤0.017; total +141%, low +100%, high +164%) and eyes open (p≤0.014; total 101%, low 96%, high 111%) had the meningitis subjects poorer stability compared with healthy controls, as revealed by the significant “Meningitis” factor in all spectral categories. Moreover, during eyes closed condition, repeated balance perturbations disturbed stability less over time in both subject groups, as shown by the significant “Period” factor in all spectral categories (p≤0.002; total −17%, low −27%, high −9%). However, with eyes open were the “Period” findings mixed. The stability was found weakly improved in the total (p = 0.004; −3%) and low frequency spectra (p = 0.024, −8%) whereas the stability was poorer in the high frequency spectra over time (p = 0.008, +4%). Furthermore, the significant interaction between “Meningitis” and “Period” factors in the total frequency spectra, indicated that the lateral stability improvement was poorer over time in meningitis subjects both with eyes closed (p = 0.029; −25% in controls vs. −8% in meningitis subjects) and eyes open (p = 0.027; −29% in controls vs. +23% in meningitis subjects).

The post-hoc analysis of data from individual tests showed that the stability in meningitis subjects was poorer in anteroposterior direction during balance perturbations compared with controls, though the group differences reached significance only with eyes open during period 3 in all spectral categories (p≤0.040) while indicated by trends at three other vibration periods, see Figure 1. In lateral direction, the post-hoc analysis confirmed poorer stability during balance perturbations in meningitis subjects compared with controls in all periods, all spectral categories and with eyes closed as well as with eyes open.

### Adaptation capacity

Healthy controls adapted significantly to the repeated balance perturbations in anteroposterior direction with eyes closed (p<0.001) and eyes open (p≤0.002) in all spectral categories (for size values see Table 4). In the same direction, the meningitis subjects did also adapt significantly in all spectral categories with eyes closed (p<0.001) but only significantly in the total and low frequency spectra (p≤0.018) with eyes open. Moreover, the quantitative improvements gained from the adaptation were lower in the meningitis subjects than in controls in all spectral categories both with eyes open and closed.

**Table 4:** Adaptive changes achieved by subjects treated in childhood for meningitis and by healthy controls, from period 1 to period 4 in the stimulation sequence.

Anteroposterior Adaptation*		Eyes Closed	Eyes Open
Healthy controls	Total	**<0.001 [**−**48%]**	**<0.001 [**−**59%]**
	<0.1 Hz	**<0.001 [**−**64%]**	**<0.001 [**−**73%]**
	>0.1 Hz	**<0.001 [**−**36%]**	**0.002 [**−**29%]**
Meningitis subjects	Total	**<0.001 [**−**33%]**	**0.018 [**−**22%]**
	<0.1 Hz	**<0.001 [**−**43%]**	**0.008 [**−**32%]**
	>0.1 Hz	**<0.001 [**−**29%]**	0.108 [−12%]
**Lateral Adaptation**			
Healthy controls	Total	**0.002 [**−**25%]**	**0.031 [**−**29%]**
	<0.1 Hz	**0.021 [**−**35%]**	**0.009 [**−**52%]**
	>0.1 Hz	**0.021 [**−**16%]**	0.266 [−1%]
Meningitis subjects	Total	**0.012 [**−**8%]**	0.867 [+23%]
	<0.1 Hz	0.695 [−19%]	0.430 [+36%]
	>0.1 Hz	**0.004 [**−**2%]**	0.501 [+9%]

*The statistical differences are presented followed by values showing the size of the changes in percent (within the squared parenthesis). A (−) represent a reduction over time and a (+) an increase over time.

The controls adapted significantly to the repeated balance perturbations also in lateral direction in all spectral categories with eyes closed (p≤0.021) and in the total and low frequency spectra (p≤0.031) with eyes open. In lateral direction the meningitis subjects did only adapt significantly in the total and low frequency spectra (p≤0.012) with eyes closed, but did not adapt at all with eyes open. The quantitative improvements gained over time were lower in meningitis subjects than in controls in all spectral categories, both with eyes open and closed. The quantitative values from tests with eyes open even suggest that the meningitis subject’s lateral stability declined over time, though these changes could not be confirmed statistically.

### Hearing

Thirty-three out of 36 subjects had normal hearing, i.e., PTA of thresholds at 0.5, 1, 2, 4 kHz (PTA_5124_) ≤20 dB HL, normal tympanograms, i.e., type Jerger’s A and normal DPOAE response (S/N ratio ≥3 dB). Two of the 3 subjects had unilateral (both right sided) hearing loss, i.e., PTA of thresholds at 0.5, 1, 2, 4 kHz (PTA_5124_) ≥90 dB HL, absent DPOAE response (S/N ratio ≤3 dB) affected ears, but bilateral normal tympanograms, i.e., type Jerger’s A. These subjects were both infected by *S. Pneumonia* and clinically did not express peripheral vestibular imbalance (left overweight) or otherwise impaired peripheral compensation. One subject had moderate unilateral (left ear) hearing impairment, i.e., PTA of thresholds at 0.5, 1, 2, 4 kHz (PTA_5124_) = 56 dB HL, absent DPOAE response (S/N ratio ≤3 dB) affected ear, but bilateral normal tympanogram, i.e. type Jerger’s A. This subject was infected by *N. Meningitides* and clinically did not express peripheral vestibular imbalance (right overweight) or otherwise impaired peripheral compensation.

### Analysis of questionnaire

The questionnaire revealed that the meningitis subjects experienced a range of symptoms (Table 5), such as unsteadiness, light-headedness (88% of the subjects) or the feeling that things around them were spinning or moving (81%). Other common symptoms were feeling visual disturbances (i.e. blurred, flickering, blind spots in the visual field) (72%), headache (75%), nausea (72%) and feeling hot or cold spells (75%).

**Table 5:** VSS questionnaire answers from the meningitis subjects (n = 36) to the question: “How often during the last 12 months have you had the following symptoms”.

VSS questionnaire answers	Between 1–12 timesper year	More than oncea month	In total*
1. Heart/chest pain?	17	4	21
2. Hot or cold spells?	17	10	27
3. Falling over?	12	3	15
4. Nausea, feeling sick?	17	9	26
5. Tense/sore muscles?	15	5	20
6. Trembling/shivering?	16	2	18
7. Pressure in the ears?	20	3	23
8. Heart pounding?	11	2	13
9. Vomiting?	12		12
10. Arms/legs feel heavy?	9	1	10
11. Visual disturbance?	17	9	26
12. Headache?	16	11	27
13. Unable to stand, walk?	4		4
14. Breathing difficulties?	9	3	12
15. Poor concentration?	13	7	20
16. Tingling, pricking?	18	6	24
17. Lower back pain?	8	8	16
18. Excessive sweating?	13	4	17
19. Feeling faint?	15		15
20. Things spinning/moving, lasting:			
- Less than 2 min	17	4	21
- 2 to 6 min	6		6
- More than 1 hour	2		2
- Whole day			0
** - In total**	**25**	**4**	**29**
21. Light-headedness/giddiness, lasting:			
- Less than 2 min	16	7	23
- 2 to 6 min	6		6
- More than 1 hour	1	1	2
- Whole day			0
** - In total**	**23**	**8**	**31**
22. Unsteadiness, lasting:			
- Less than 2 min	13	2	15
- 2 to 6 min	1		1
- More than 1 hour			0
- Whole day			0
** - In total**	**14**	**2**	**16**

*Subjects not grouped for the question in the table had not experienced the symptoms described during the period.

The correlation analysis between the questionnaire and assessed postural stability revealed numerous significant relationships, see Table 6. In total, 43 significant correlations were found, all of them with an R-value showing a relationship between reported more subjective problems and findings of poorer stability confirmed by the posturography tests. Some subjective symptoms commonly found related to poorer stability were: feeling heaviness in the arms and legs; feeling light-headed; unsteady; that things around them were spinning or moving and feeling headache.

**Table 6:** Significant correlations between answers to the questionnaire and postural stability in anteroposterior and lateral direction with eyes closed and eyes open.

Correlation questionnaire answers vs.postural stability*		Quiet Stance	Period 1	Period 2	Period 3	Period 4
Antero-posteriorEyes Closed	Pressure in the ears?				**0.037** **[0.348]**	**0.023** **[0.379]**
	Arms/legs feel heavy?	**0.009** **[0.427]**				
	Headache?	**0.022** **[0.382]**			**0.035** **[0.352]**	
	Feeling faint?	**0.021** **[0.383]**				
	Things spinning/movinglasting:					
	- 2 to 6 min				**0.023** **[0.377]**	**0.031** **[0.407]**
	Light-headedness/giddinesslasting:					
	- Less than 2 min				**0.041** **[0.342]**	**0.026** **[0.372]**
	Unsteadiness lasting:					
	- Less than 2 min	**0.038** **[0.348]**				
Antero-posteriorEyes Open	Arms/legs feel heavy?			**0.005** **[0.456]**		**0.001** **[0.515]**
	Headache?				**0.035** **[0.353]**	**0.009** **[0.431]**
	Tingling, pricking?					**0.034** **[0.354]**
	Things spinning/movinglasting:					
	- 2 to 6 min				**0.033** **[0.356]**	
	Light-headedness/giddinesslasting:					
	- Less than 2 min					**0.026** **[0.371]**
	Unsteadiness lasting:					
	- Less than 2 min					**0.034** **[0.354]**
Lateral EyesClosed	Pressure in the ears?	**0.044** **[0.337]**			**0.037** **[0.349]**	
	Arms/legs feel heavy?	**0.013** **[0.411]**				
	Feeling faint?	**0.049** **[0.330]**	**0.032** **[0.358]**			
	Things spinning/movinglasting:					
	- 2 to 6 min	**0.047** **[0.333]**				
Lateral EyesOpen	Arms/legs feel heavy?	**0.003 [0.484]**	**0.005** **[0.457]**	**0.005** **[0.457]**	**0.031** **[0.361]**	**0.022** **[0.381]**
	Visual disturbance?			**0.025** **[0.373]**		
	Headache?		**0.030** **[0.362]**	**0.015** **[0.401]**		**0.030** **[0.363]**
	Feeling faint?		**0.017** **[0.397]**			
	Things spinning/movinglasting:					
	- 2 to 6 min					**0.045** **[0.337]**
	Light-headedness/giddiness lasting:					
	- Less than 2 min		**0.034** **[0.354]**	**0.013** **[0.412]**	**0.005** **[0.461]**	**0.042** **[0.341]**
	Unsteadiness lasting:					
	- Less than 2 min			**0.021** **[0.384]**	**0.016** **[0.398]**	**0.019** **[0.389]**

*The correlation R-values are presented within the squared brackets. A positive R-value represents that a poorer stability are related to more frequent subjective symptoms among the meningitis subjects. Correlations were performed when the symptoms were recognized by more than 5 subjects (i.e., >17%).

### Age at time of treatment and Time elapsed after end of treatment

A significant correlation (R = -0.671, p<0.001) was found between Age at time of treatment and Time elapsed after end of treatment. This means that the individual roles of these factors might be difficult to separate in a statistical evaluation. However, none of these variables were correlated with recorded postural stability for any test condition, period or sway direction. Only one relationship between an answer in the questionnaire and Age at the time of treatment, i.e., those that had received treatment at older age reported more problems with feeling pressure inside the ears (R = 0.336, p = 0.045). There was no relationship between any answers in the questionnaire and Time elapsed after end of treatment.

## Discussion

This study revealed that adult survivors of childhood bacterial meningitis infection suffer from a variety of long-term deficits that affects postural control and CNS ability to improve stability through adaptation and vision. These deficits are at a scale causing meningitis subjects on average to use 90–140% more energy on postural control during quiet stance with eyes open, which makes them clinical relevant to diagnose. Moreover, the safety margin for falls decrease, though the deficits observed in this study would probably cause falls only associated with other heavily straining conditions. When summarizing the findings, the properties of the deficits found are complex and appear influenced by context: 1) Meningitis subjects had poorer stability compared with controls both during the quiet stance and when exposed to balance perturbations as recorded in anteroposterior and lateral directions. That said, the meningitis subjects handled quite well the balance perturbations during the first 1–2 minutes in anteroposterior direction, but thereafter seemed poorly able to handle the sustained perturbation; 2) The stability deterioration found in the meningitis group was most marked with eyes open, suggesting that the ability to collect, analyze or utilize visual information to enhance stability might be compromised; 3) The ability to adapt to the balance perturbations was decreased in the meningitis subjects, though most prominently with eyes open and in lateral direction; 4) Meningitis subjects reported a host of subjective symptoms associated with dizziness, visual impairments and vertigo. The most common symptoms were feeling unsteady, light-headedness, spinning sensation and visual disturbances (i.e. blurred, flickering, blind spots in the visual field); 5) There were numerous correlations between self-reported symptoms and poorer stability measured using posturography, suggesting a close relationship between feeling certain symptoms and having objectively decreased stability performance.

Structural changes in the CNS and inner ear due to bacterial meningitis are known, where the parenchymal damaging factors are cytotoxic and vasoactive edema with increased ICP and leukocyte infiltration. These pathological processes are mediated by the dual effects of overwhelming inflammatory reaction and direct effects of bacterial toxins [33]. Moreover, ossification of the cochlea and semicircular canals has been described [6]. Since these as important afferent information links for postural control may such deficits contribute to stability disturbances [24]. Neuronal apoptosis in dentate gyrus, an important part of the hippocampus, have been detected on autopsies of those dying from bacterial meningitis [34]. Hippocampus plays an important role in spatial learning and memory and is influenced by vestibular information [35]. As animal studies confirm the neuronal apoptosis in bacterial meningitis and demonstrate as well its long term effects [36], [37] it is probable that some of the balance disturbance and adaptations findings in our study might be linked through these CNS areas. As 33 out of 36 subjects in our study had normal hearing, and the remaining 3 did not express vestibular end organ impairment on clinical tests, one may draw the conclusion that their inner ears are intact and therefore not contributing to the postural control disturbances, which are then presumably of CNS origin.

### Postural control and influence of age at meningitis infection and past time after the infection

Assessed postural stability provides a clinical estimation of the risk of falling but also offers an investigative window into changes of the neurophysiological mechanisms [38]. The present study revealed that meningitis subjects, on average 12.5 years after the infection, had significantly decreased stability in both quiet and perturbed standing compared with controls. Given the properties of the findings and that the assessments were done so long time after the subjects had their meningitis infection, decreased postural control appears to be a common long-term aftermath from bacterial meningitis infection. When time elapsed after treatment was correlated with recorded stability, no relationship was found, indicating a recovery processes, i.e., that those assessed a longer time after the infection performed better in the posturography tests. That said, correlation analysis is not the ideal method to determine whether there is a recovery process. However, in this case, the difficulties to objectively compare stability performances of young children with adults, present research conditions preventing proper pair-wise comparisons to determine the presence of any recovery process.

The developmental state of younger subjects at the time of meningitis infection, i.e. age at time of treatment, might be an important factor influencing the severity of the long-term impairments experienced later in life as an adult. The fundamental mechanisms behind postural control (e.g. sensorimotor development and functional ability) are formed and modified during childhood motor development [39]–[41]. Thus, it can be hypothesized that younger children are more vulnerable to long-term deficits from meningitis because the postural control system is still under development. However, in this study we did not find statistical evidence suggesting that those treated for meningitis at young age had more severe postural control deficits compared with the ones treated later or that they reported more subjective symptoms in the questionnaire.

Postural instability was observed both in a lateral and anteroposterior direction in the meningitis subjects. However, several statistical findings suggest that the lateral stability was proportionally more severely affected by the meningitis infection than the anteroposterior stability, a noteworthy observation also by that the balance perturbations used mostly induced increased movements in anteroposterior direction. The stability in lateral direction has often received less attention in earlier clinical research reports, possibly because the lateral movements typically are smaller than anteroposterior movements. However, stability limits are not determined by the size of the movement alone, but also by how large the movements are in that direction relative to the stability constraints set by the biomechanical design of the human body, i.e., maximum leaning possible in a direction before falling.

### Adaptation and meningitis

Postural control ability to adapt and habituate its balance control processes, making them more versatile and resilient when handling stability issues based on prior experiences, is important for human movement control, fall prevention [20], [42] and performance enhancement during various human activities [43], [44]. The controls were able to adapt to the balance perturbations evoked by the vibratory stimulation, but the meningitis subjects had problems to learn to handle the effects of the balance perturbations, especially its manifestation in lateral direction with eyes open. Postural control adaptation including optimizing integration of information from the visual, vestibular and somatosensory receptors and motor coordination are complex processes, especially when information from any of the sensory systems is not reliable [45]. Meningitis subjects are frequently reported to have reduced cognition and concentration, indicating poor attention [9]. One possible reason for poorer stability and adaptation in meningitis subjects in the lateral direction could therefore be that limited attention or cognitive resources forced a “severe stability threats - first adaptation priority” principle to be applied. Thus, meningitis subjects may have focused the attention and adaptation capacity they had where it appeared to matter the most, i.e., in the direction of biggest stability threat, which in the present case was anteroposterior direction because the stability was disturbed the most in this direction by the balance perturbations. Similar limitations in capacity to adapt the stability control in more than one direction has been observed under severe acute alcohol intoxication [46].

### Vision and meningitis

Although vision improved stability in all subjects, meningitis subjects were particularly with eyes open less stable than controls in lateral direction and had poorer motor control adaptation than controls. In addition, high frequency activity was significantly increased in meningitis subjects and more so in lateral direction than in anteroposterior direction, but unlike controls, high frequency activity was not reduced well with eyes open. These findings suggest that bacterial meningitis may affect the visual system and its contribution to postural control. Moreover, the frequent reports in the questionnaire of experiencing visual disturbances is another indication of that the visual system and its ability to provide accurate information might be compromised in meningitis subjects. Hence, although visual information improves stability, this additional information seemed to be used far less effective to enhance postural control in those treated for meningitis, which advocate for more research to investigate whether the ability to collect, analyze or utilize visual information for postural control might be compromised by bacterial meningitis.

### Questionnaire symptoms and postural control

Meningitis subjects reported many subjective symptoms associated with dizziness, vertigo and visual impairments. The most common symptoms found were feeling unsteadiness, light-headedness and the feeling that things spin or move and visual disturbances (i.e. blurred, flickering, blind spots in the visual field) (Table 5). Childhood meningitis has also various other known sequelae such as sensorimotor deficits including hearing and visual loss and motor dysfunction [9], [10]. Unfortunately, the control material used in this study was not screened with the questionnaire, which is a shortcoming. However, although it is likely that also healthy people feel some of these symptoms (e.g., headache) the high frequency of feeling such symptoms in the meningitis group and the numerous significant correlations to recorded stability issues suggest that these symptoms are not random in nature but related to the childhood meningitis infection. Moreover, the methodical distribution of the significant questionnaire correlations to the stability recorded both in anteroposterior and lateral direction suggest that the stability deficits have a structure and a systematic relationship to a higher rate of feeling specific subjective symptoms. For example, a large number of significant correlations between subjective symptoms and quiet stance anteroposterior and lateral stability, were found but with one exception only when standing with eyes closed. Moreover, in anteroposterior direction and both during tests with eyes closed and open, the relationship between having poorer stability and more subjective symptoms became apparent first during the last perturbation phases 3 and 4, which usually is when attention loss and fatigue starts to have an impact. In lateral direction, relationships between subjective symptoms and poorer stability were present throughout the entire posturography tests while standing with eyes open but occurred only twice, seemingly random, with eyes closed. Hence, given the diversity of the co-occurring long-term symptoms experienced, impairments caused by having meningitis in childhood may not be restricted to one function only, but may cause a long-term decline of several neurophysiological functions.

Although our results are in accordance with recent publications [24] there are certain limitations to this study. Firstly, the vestibular end-organ function was not objectively assessed in this study. The end-organ function was however evaluated indirectly through clinical headshake and head thrust tests, which revealed no peripheral loss or imbalance of vestibular information. Secondly, the significant R-values in the correlation analysis, starting from R = 0.330, could indicate that the relationships found were significant but also of relatively low functional importance. However, when evaluating R-values, it is important to consider the mathematical conditions under which these values are produced and whether the analyses might be influenced by factors known to cause underestimation of correlation strengths. Hence, given the properties of the data sets in this study, i.e., data sets with non-normal distributions, the high relationship complexity and the heterogenic limitations, the true correlation strengths may not be precisely reflected by the size of the R-values.

To summarize, we believe that our study adds to the increasing pool of knowledge on long-term complications of meningitis in childhood. It is important to recognize that postural control and CNS adaptation might be impaired even in patients with no hearing sequelae, as these complications can be improved markedly in most cases with effective and targeted interventions.
